# Supplementary material for: Non-Traditional Physical Education Classes Improve High School Students’ Movement Competency and Fitness: A Mixed-Methods Program Evaluation Study
Source: Int J Environ Res Public Health. 2023 May 22;20(10):5914. doi: 10.3390/ijerph20105914 (PMC10218483; doi:10.3390/ijerph20105914)
Supplement: Supplementary file 1 [file ijerph-20-05914-s001.zip › ijerph-2332280-supplementary.docx]

Supplemental Materials

**Table S1.** Example workouts from the CrossFit Physical Education class.

| **Day** | **CrossFit Class Workout Structure** | **Workout Details** |
| --- | --- | --- |
| 1 | -Timed  -Gymnastics Couplet  -High Rep  -Medium Duration | For Time: 27-21-15-9 Burpees & V-ups |
| 2 | -Timed  -Weightlifting and Monostructural Triplet  -High Repetition  -Medium Duration | 6 rounds for time of:  60 Double Unders  Dumbbell Farmers Carry, 50/35 lbs, 80 m  Dumbbell Lunge, 50/35 lbs, 10 m |
| 3 | -Gymnastics Skill work for Quality (not timed)  -Gymnastics Couplet (for time). | A). Every 1 min for 5 mins: 5 Wall Walks  B). 5 rounds for time of: Handstand Hold, 1 min and 21 Toes-to-bars |
| 4 | -Timed  -Monostructural and WeightliftingTriplet  -Medium Rep, range, duration, and loading. | For time:  Double Unders, 1 min  21 Front Squats, 95/65 lbs  21 Push Press, 95/65 lbs  Double Unders, 1 min  15 Front Squats, 95/65 lbs  15 Push Press, 95/65 lbs  Double Unders, 1 min  9 Front Squats, 95/65 lbs  9 Push Press, 95/65 lbs |
| 5 | Heavy Day | Clean & Jerk 2-2-2-2-2-2-2  -Use the heaviest weight you can for each set.  Rest as needed between sets. |
| 6 | -Timed  -Gymnastics Couplet  -High repetition and medium duration. | Teams of 2 - 10 rounds for time of:  Partner A:  10 Strict Pull-ups  10 Burpees  Partner B:  10 Strict Pull-ups  10 Burpees |
| 7 | -AMRAP^1^  -Weightlifting & Gymnastic Couplet  -Benchmark  -Medium, Reps, Duration & Loading. | “The Chief” Benchmark workout  For 5 cycles:  AMRAP in 3 mins of:  3 Power Cleans, 135/95 lbs  6 Push-ups  9 Air Squats  Rest 1 min between each cycle.  For each cycle restart the AMRAP. |
| 8 | -AMRAP Gymnastic Triplet  -High Rep, Medium Duration, and no loading. | “Cindy” Benchmark Workout  Complete as many rounds as possible in 20 mins of:  5 Pull-ups  10 Push-ups  15 Air Squats |
| 9 | HeavyDay | Back Squat 3-3-3-3-3  -Use the heaviest weight you can for each set.  Rest as needed between sets. |
| 10 | CrossFit Games Open 22.2 - Scaled Teens 14-15:  -For time  -Weightlifting and Gymnastics Couplet  -light loading, medium duration, high rep | 1-2-3-4-5-6-7-8-9-10-9-8-7-6-5-4-3-2-1 reps of:  Deadlift, 75/55 lbs  Bar Facing Burpee*  Time cap: 10 mins  *Stepping over the bar is allowed. |

^1^ AMRAP – as many rounds as possible.

**Table S2.** Example workouts from the Weight Training for Athletic Performance Physical Education class

| **Day** | **Weightlifting Class Workout Structure** | **Workout Details** |
| --- | --- | --- |
| 1 | -Olympic Lifting Technique Day  -Below 60% Loading | 16 minute EMOM^1^: 1 Power Clean & Push Jerk + Hang Squat Clean & Split Jerk, pick load (alternate movements each minute) |
| 2 | -Lower Body Hinge/Upper Body Pull (rest as needed). | 5-5-5-10 reps  Deadlift, 80% 1RM^2^  Pull-ups (banded, bodyweight, or loaded).  *60-120s rests. |
| 3 | -Upper Body Push/Pull  -Giant Set with 2-3 minute rests between sets. | 4 sets for quality of:  8 Bench Press (70-75%)  10 Banded Push-ups  10 Dumbbell Pullovers |
| 4 | -Upper Body Pull/Push  -Lower Body Push  -Olympic Lifting | Clean & Jerk 2-2-2-2-2-2-2 (3 minute rests) |
| 5 | -Lower Body Push  -RFD^3^ Training | 4 rounds for quality of:  10 Back Squats  6 Side-to-Side Skater Jump into Vertical Jump  -Rest 3 minutes between Sets |
| 6 | -Upper Body Push  -Bench Press Pyramid | Bench Press 8-6-4-3-2-1-6 (2-3 minute rests between sets). |
| 7 | -Lower Body Push variation | Box Squats 5-5-5-5-5  Use the heaviest weight you can for each set.  Rest 2-3 minutes between sets |
| 8 | -Power & Speed Development Day.  -Olympic Lifting | Hang Power Snatch 2-2-2-2-2-2-2  (85-90%).  -Rest 3 mins between sets. |
| 9 | -Upper Body Accessory work and hypertrophy.  -Emphasis on slow eccentric movements with lighter weight. | 4 rounds for quality of:  6 Floor Press  8 Single Arm Dumbbell Rows  12 Banded Push-ups  12 Bench Dips  12 Dumbbell Chest Flies  12 Hammer Curls |
| 10 | -Lower Body Squat/Hinge  -Upper Body Stability  -Olympic Lifting. | Snatch Grip Deadlift 3-3-3-3-3 (85-90%)  Use the heaviest weight you can for each set.  Rest 3 minutes between sets. |

^1^ EMOM – every minute on the minute, ^2^ RM – repetition maximum, ^3^ RFD – rate of force development.
